# Chemical Stability of a Chinese Herbal Spirit Using LC-MS-Based Metabolomics and Accelerated Tests

**DOI:** 10.3389/fphar.2022.857706

**Published:** 2022-03-07

**Authors:** Yan Hu, Zhe Wang, Jiayue Liu, Wen Yang, Qiang Yang, Yuan-Cai Liu, Qiu-Yun You, Xiao-Jia Chen, Jian-Bo Wan

**Affiliations:** ^1^ State Key Laboratory of Quality Research in Chinese Medicine, Institute of Chinese Medical Sciences, University of Macau, Macao, China; ^2^ State Key Laboratory Breeding Base of Systematic Research, Development and Utilization of Chinese Medicine Resources, School of Pharmacy, Chengdu University of Traditional Chinese Medicine, Chengdu, China; ^3^ Hubei Provincial Key Laboratory for Quality and Safety of Traditional Chinese Medicine Health Food, Jing Brand Co., Ltd, Wuhan, China; ^4^ Pharmacy School, Hubei University of Chinese Medicine, Wuhan, China

**Keywords:** chinese herbal spirit, metabolomics, accelerated test, quality control, anti-fatigue

## Abstract

As a prevalent medicinal liquor among Chinese people, a type of Chinese herbal spirit from Jing Brand Co., Ltd (CHS-J) is a newly developed health beverage with the health functions of anti-fatigue and immune enhancement. The researchers from the enterprise found that the contents of several components in CHS-J samples have been significantly decreasing during the stated storage period, as detected by the HPLC-UV method, which would make a great challenge for quality control of CHS-J. Furthermore, the chemical stability of CHS-J during the storage period is greatly challenged affected, especially in the environment of high temperature and light exposure. To systematically reveal the unstable components and promote the quality control of CHS-J, the chemical stability of CHS-J during the shelving storage period was characterized by the UPLC/Q-TOFMS-based metabolomics approach. First, the targeted and untargeted metabolomics approaches discovered the significantly changed components in CHS-J samples produced in different years. Furthermore, the accelerated tests of newly produced CHS samples and several authorized standards were conducted to validate the above results and elucidate the possible mechanisms underlying these chemical changes. Moreover, these chemical changes during the storage period had little influence on the anti-fatigue effect of CHS-J samples. These findings will offer new insight into the understanding of the chemical stability of CHS-J and will facilitate the quality control of CHS-J.

## Introduction

Chinese herbal spirit (CHS) is an alcoholic beverage in which several precious herbs are commonly immersed. CHS has become increasingly popular among Chinese people in past decades due to its nourishing and tonic activities. A type of CHS from Jing Brand Co., Ltd (CHS-J) is a newly developed health beverage, which is brewed by modern bioengineering technology with a faint-scented Xiaoqu liquor and the extracts of several Chinese herbal medicines, such as *Astragalus membranaceus* (Fisch.) Bge. [Fabaceae; Astragali Radix], *Epimedium brevicornu* Maxim [Berberidaceae, Epimedii Folium], *Cistanche deserticola* Ma [Orobanchaceae; Cistanches Herba], *Lycium barbarum* L. [Solanaceae; Lycii Fructus], and *Curculigo orchioides* Gaertn. [Hypoxidaceae; Curculiginis Rhizoma] (Liu et al., 2018). These herbal medicines are carefully composited under the guidance of traditional Chinese medicine theory, and several of them are edible Chinese Medicines and used as foods. CHS-J has been shown to exert anti-inflammation (Feng et al., 2013), health-care functions of immune enhancement (Liu et al., 2018), and anti-fatigue (Liu et al., 2018). CHS-J contained enormous and complex components with great structural diversity and a wide range of concentration distribution, including flavonoids, saponins, alkaloids, phenylethanoids, coumarins, anthraquinones, and volatile oil, etc. (Hu et al., 2021), which were blended in 35% (*v*/*v*) of the base liquor. Thus, the chemical stability of CHS-J during the storage period is greatly challenged, especially in the environment of high temperature and light exposure. The researchers from the enterprise found that the contents of several components in CHS-J have significantly been decreasing during the storage period, as detected by the HPLC-UV method, which would make a great challenge for quality control of CHS. Moreover, whether these changes during the storage period influence the function of CHS-J (such as the anti-fatigue effects) remains unclear.

As an emerging omics platform, metabolomics allows to comprehensively characterize entire small molecules (M.W. < 1000 Da) in the biological system and their dynamic changes in response to internal and external factors (Wan et al., 2013). Metabolomics emphasizes the whole system rather than individual parts, and this feature is in accordance with the natural characteristics of Chinese Medicines, such as the holistic and dynamic nature (Wang et al., 2011a). In the past years, the metabolomics approach has been widely employed for quality evaluation and discrimination of Chinese Medicines (Chan et al., 2007; Lee et al., 2009; Toh et al., 2010; Wang et al., 2011b; Sun and Chen, 2011; Xiang et al., 2011; Kim et al., 2012; Yang et al., 2012). It has also been used to monitor the changes in both chemical components and their concentrations in a given Chinese medicine that is derived from various species (Lee et al., 2009; Xiang et al., 2011), different geographical areas (Sun and Chen, 2011), processing methods (Chan et al., 2007; Toh et al., 2010), and growing years (Yang et al., 2012). Thus, metabolomics offers a promising approach for the in-depth understanding of the chemical stability of CHS-J during the shelving storage period. Ultra-high performance liquid chromatography/quadrupole time-of-flight mass spectrometry (UHPLC/Q-TOFMS) is the most popular and desirable analytical platform for mapping the chemical components in the biological sample due to its high throughput, high sensitivity, and depth of coverage (Xia et al., 2020). Therefore, in the current study, the chemical stability of CHS-J during the shelving storage period was characterized by the UPLC/Q-TOFMS-based metabolomics approach. First, the significantly changed components in CHS-J samples produced in different years were discovered by targeted and untargeted metabolomics approaches. Second, the accelerated tests of newly-produced CHS samples and authorized standards were conducted to validate the above results and elucidate the possible mechanisms underlying these chemical changes. Furthermore, the anti-fatigue effects of CHS-J samples with different storage periods was also conducted and compared.

## Materials and Methods

### Materials and Chemicals

CHS-J specimens produced by five different years, i.e., 2014, 2015, 2016, 2018, and 2020 (*n* = 20) and 35% (*v*/*v*) of base liquor of CHS-J were kindly provided by Jing brand Co. Ltd (Hubei, China), and the company declined to disclose the detailed information, such as the amount of each herb and the extraction procedure, as a trade secret. All specimens were stored at room temperature and light- protected environment at the Institute of Chinese Medical Sciences, University of Macau, Macao. Thirteen reference compounds, including Z-ligustilide, Baohuoside Ⅰ, Sagittatoside B, Epimedin B, Echinacoside, 2″-O-rhamnosyl Icariside II and Magnoflorine, were purchased from Baoji herbest Bio-Tech Co. Ltd (Shaanxi, China). Their purities were over 98%, as detected by HPLC-UV. Acetonitrile (HPLC-grade) and formic acid (HPLC-grade) were obtained from Merck (Darmstadt, Germany) and Aladdin Industrial Inc. (Shanghai, China), respectively. Deionized water was prepared by a Millipore Milli-Q water purification system (Bedford, MA, United States). A drying and heating oven (ED260 model, BINDER GmbH, Tuttlingen, Germany) and constant climate chambers (KBF 240 model, BINDER GmbH) were used in temperature-accelerated and light-accelerated tests, respectively.

### Sample Preparation

Approximate 1 ml of CHS-J samples was mixed with the equivalent volume of acetonitrile. The solution was vortexed for 1 min and then centrifuged at 4^°^C (14,800 rpm, 20 min). The supernatant was filtered through a 0.22 μm polytetrafluoroethylene filter (Millipore, Bedford, MA, United States) prior to the UHPLC-MS analysis. An equal volume (10 μl) of each CHS-J sample was mixed to generate a pooled QC sample, which offers a representative “mean” sample consisting of all analytes. The QC sample was analyzed 6 times at the beginning and end of the sequence, and then every 10 tested CHS-J samples to evaluate the system stability and method reproducibility.

### Untargeted Metabolomics Analysis

After sample preparation, CHS-J samples were analyzed by UHPLC-Q-TOFMS. Chromatographic separation was conducted on a Waters ACQUITY UPLC BEH C_18_ column (100 × 2.1 mm, 1.7 μm) with column temperature at 35°C. The analytes were subjected to gradient elution chromatography with a mobile phase composed of 0.1% formic acid (*v*/*v*) in water (phase A) and 0.1% formic acid (*v*/*v*) in acetonitrile (phase B). The solvent gradient elution program was used as follows with the flow rate of 0.3 ml min^−1^: linear gradient from 5 to 40% B (0–15 min), 40–70% B (15–18 min), 70-100% B (18–20 min),: isocratic 100% B (20–22 min), 100–5% B (22–22.1 min), 5% B (22.1–25 min). The sample injection volume was 2 μl, and the autosampler temperature was maintained at 4°C. The electrospray ionization (ESI) source with MS^E^ continuum acquisition mode was adopted to collect mass spectra in positive and negative ion modes. The key MS parameters were set as follows: MS range, *m/z* 50 to *m/z* 1200; capillary voltage, +3.0 kV in ESI+ and -2.5 kV in ESI-; cone voltage (CV), 40 V; cone gas flow, 50 L h^−1^; desolvation gas flow, 600 L h^−1^; source temperature, 100 °C; desolvation temperature, 250 °C. A lock-mass calibrant of leucine-enkephalin (200 ng/ml) was continuously infused at flow-rate of 50 μl/min via a lock spray interface, generating the reference ions ([M+H]^+^ = 556.2771, [M−H]^−^ = 554.2615) for mass correction during the analysis.

### Targeted Metabolomics Analysis

To determine the levels of seven changed compounds highlighted in untargeted metabolomics, including Z-ligustilide, baohuoside Ⅰ, sagittatoside B, epimedin B, echinacoside, 2″-O-rhamnosyl icariside II and magnoflorine, an ACQUITY™ UPLC system coupled with a Xevo TQD triple-quadrupole tandem mass spectrometry (QQQ-MS/MS, Waters Co., Manchester, United Kingdom) were used to quantitatively determine these components in CHS-J samples. Chromatographic separation was achieved on the identical BEH C_18_ column using the same mobile phases. The flow-rate was set at 0.3 ml min^−1^ with the following solvent gradient: 5–40% B (0–12 min), 40–100% B (12–16 min), 100% B (16–18 min), and the re-equilibrated by 5% B for 3 min. Data acquisition was conducted on a Xevo TQD QQQ-MS equipped with an ESI using multiple reaction monitoring (MRM) mode. MS was operated in the positive mode to obtain satisfactory MS response for seven investigated analytes. The optimized MS parameters were depicted as follows: capillary voltage, +3.5 kV; source temperature, 140°C; desolvation gas flow and temperature, 650 L h^−1^, and 350°C; cone flow, 50 L h^−1^. The ion transitions, cone voltage (CV), and collision energy (CE) for each compound were optimized and shown in Supplementary Table S1.

### Accelerated Tests of CHS-J Sample and Reference Standards

The chemical difference in CHS-J samples produced in different years might be attributed to the unstable components and the chemical variation of herbal medicines used in different years. To further confirm the instability of components in CHS-J sample during storage, the accelerated tests of the newly produced CHS-J (the year 2020) were conducted. In the temperature-accelerated tests, 100 ml of CHS-J samples (*n* = 8) were placed in clean glass bottles with airtight rubber seal flip caps and maintained at constant 60 ± 2°C and shield from light. 0.5 ml of liquor from each bottle was collected every 2 weeks and continuously collected for 8 weeks. In light-accelerated tests, 100 ml of CHS-J samples (*n* = 8) were exposed at the light (5500 ± 500 Lux, 50% R.H., 25°C), 0.5 ml of liquor was collected every 3 until 12 weeks. All collected liquor samples were stored at −80°C until analysis.

To reveal the potential degradation pathways, the selected reference standards were also subjected to temperature-accelerated and light-accelerated tests as described above. The reference standards were accurately weighed and dissolved in 35% (*v*/*v*) of base liquor individually at the final concentration of 20 μg ml^−1^ with 3 replicates. 0.5 ml of solution from each bottle was collected weekly. All collected samples were stored at −80°C until UHPLC-Q-TOFMS analysis.

### Anti-Fatigue Study

#### Animal treatment

After 7-day acclimatization, a total of 60 male Kunming mice were randomly divided into the following three groups (*n* = 20 per group): control group (normal saline); 2014-year group (15 ml/kg/days CHS-J samples produced in 2014); 2020-year group (15 ml/kg/days CHS-J samples produced in 2020). Each mouse was orally administered with the CHS-J samples for 14 consecutive days. The health status of the mice was observed each day. According to the Technical standards for testing and assessment of health food (2003 edition), the exhaustive swimming time, hepatic glycogen level, muscle glycogen level, blood lactic acid level, urea nitrogen level, and lactic dehydrogenase level were tested to estimate the anti-fatigue effects (Ministry of Health of the People's Republic of China, 2003).

#### Exhaustive Swimming Test

In brief, after 30 min of last intragastric administration, a lead sheath, weighing 5% of the mouse’s body weight, was tied to the root of the mouse tail. Four tempered glass pools (50 × 40 × 40 cm) were filled with water to a depth of 30 cm. The mice (10/group) were dropped into the water. The swimming time (from dropping into the water to sinking underwater for over 10 s) was recorded. The water temperature was 25 ± 1°C.

#### Determination of Hepatic Glycogen Level and Muscle Glycogen Level

The mice (20/group) were killed by cervical vertebral dislocation, and their liver and muscle tissues were extracted for further analysis. Since glycogen in the liver tissues is unstable and loses activity quickly *in vivo*; thus, 100 mg of liver tissue from each mouse was weighed, cleaned using normal saline, dried with filter paper, and then diluted in lye immediately. The anthrone colorimetric method was adapted to estimate the quantity of hepatic glycogen and muscle glycogen.

#### Determination of Blood Lactic Acid Level, Urea Nitrogen Level, and Lactic Dehydrogenase Level

The mice (20/group) were forced to swim in pools for 90 min without weight loading. After a 60-min resting period, blood was sampled from eyes and collected in tubes containing heparin. Plasma samples were collected by centrifugation for 10 min at 3,800 rpm. The blood lactic acid levels were measured using blood lactic acid assay kits, and the concentrations of urea nitrogen were analyzed using urea assay kits. The lactic dehydrogenase activity in plasma was measured by the automatic biochemical analyzer.

## Statistical Analysis

The data acquired in UHPLC-Q-TOFMS analysis were processed by Progenesis QI software (Nonlinear Dynamics, Newcastle, United Kingdom) for peak picking and alignment. The processed data were subsequently analyzed by SIMCA-P software (version 14.1, Umetrics, Umeå, Sweden) for multivariate statistical analysis. The contents and relative peak areas of investigated analytes were analyzed by GraphPad Prism (version 6.0, San Diego, CA, United States) to visualize the variation in different groups. The levels of analytes were expressed as the mean ± standard deviation (S.D.), and a *p*-value of less than 0.05 was considered statistically significant.

## Results and Discussion

### Method Validation of UPLC/Q-TOFMS

**FIGURE 1:**
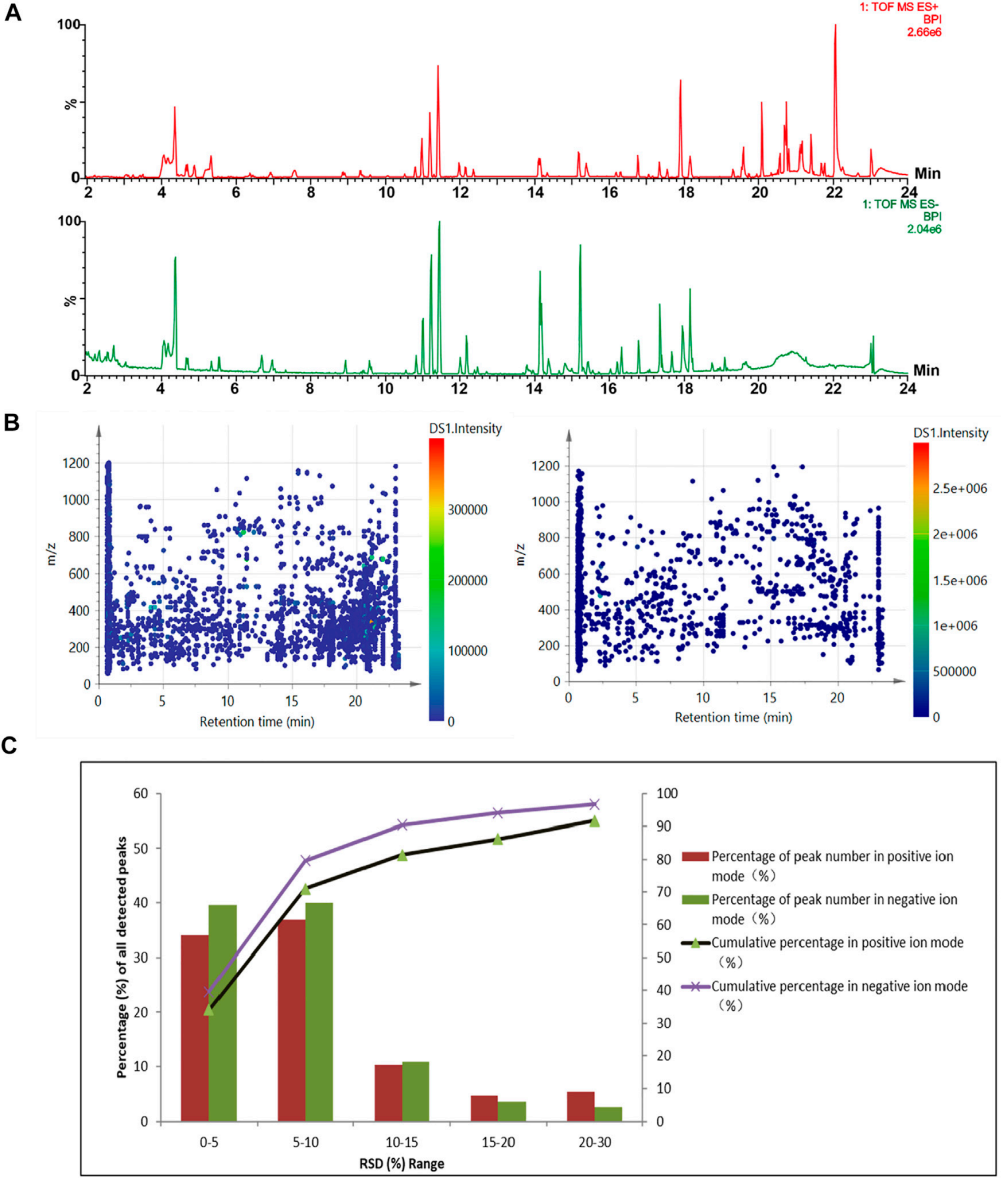
**(A)** Representative base-peak ion (BPI) chromatograms of QC samples detected in the positive (up) and negative (down) ion modes. **(B)** The distribution of the detected ions. **(C)** RSD (%) distribution of the detected peaks in the QC sample.

Due to containing several herbal extracts and the base liqueur, the CHS-J is a highly complex matrix consisting of numerous organic compounds with a wide range of concentrations. Under the optimized chromatographic and MS conditions, the major components in CHS-J were well separated and detected in both positive and negative ion modes. The representative base peak intensity (BPI) chromatograms of the QC sample are illustrated in Figure 1A. A pooled QC sample was used for the method validation of UHPLC/Q-TOFMS. The raw data acquired was pre-treated using Progenesis QI for peak picking and alignment. After “80% rule” processing, 2775 and 1143 variables were generated from the QC samples detected in positive and negative ion modes, respectively. These ions are fairly evenly distributed in whole chromatograms (Figure 1B). Furthermore, the relative standard deviation (RSD) distribution of these ions in QC samples was commonly used to evaluate the stability and reproducibility of untargeted metabolomics analysis. As shown in Figure 1C, 91.7 and 96.8% of the variables with RSD less than 30% were observed in positive and negative ion modes, respectively, along with 71.2 and 79.9% of the ions with RSD ≤10%, which demonstrated the excellent stability and reliability of the UPLC/Q-TOFMS method for untargeted metabolomics.

### Metabolomics Analysis of CHS-J Samples With Different Storage Years

**FIGURE 2:**
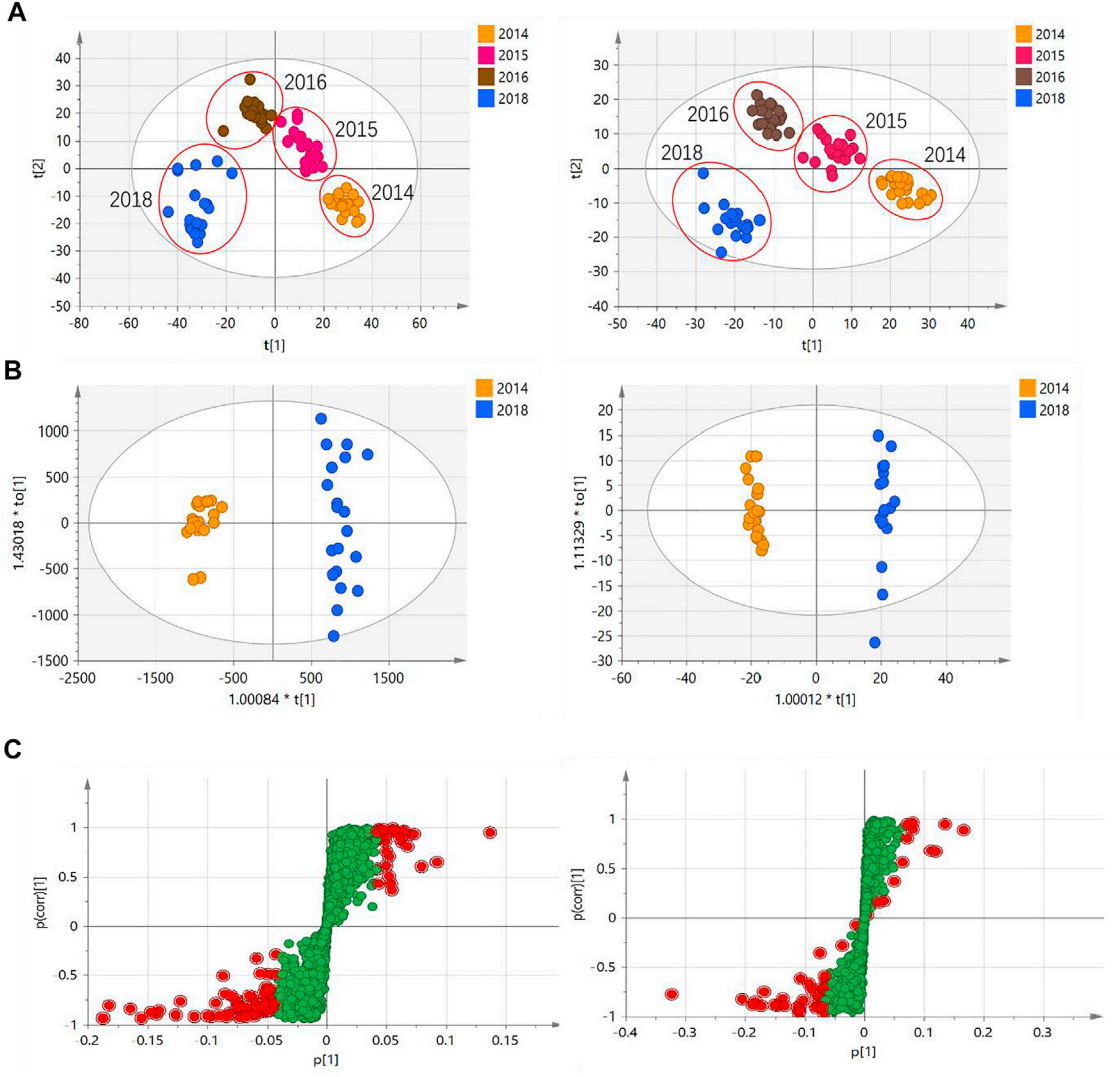
**(A)** PLS-DA score plots of CHS-J samples with different production years in positive (left) and negative (right) ion modes. **(B)** OPLS-DA score plots and **(C)** their *S*-plots of CHS-J samples produced in 2014 and 2018 detected in positive (left) and negative (right) ion modes.

To characterize the chemical differences across CHS-J samples with different storage years, CHS-J samples produced by 2014, 2015, 2016, and 2018 were used for untargeted metabolomics analysis. After data preprocessing, the partial least squares discriminant analysis (PLS-DA), a supervised multivariate data analysis, was constructed. Sevenfold cross-validation was conducted to validate the quality of the PLS-DA model. The parameters R^2^ and Q^2^ represent the interpretability of the variable and the model’s predictability, respectively. The cumulative values of R^2^X, R^2^Y, and Q^2^ were close to the optimal value of 1.0, indicating the established models with excellent predictive capability and fitness (Wan et al., 2013). As depicted in Figure 2A, all tested CHS-J samples from four different production years were clustered in PLS-DA score plots in both positive (R^2^X = 0.596, R^2^Y = 0.964, Q^2^ = 0.943) and negative (R^2^X = 0.702, R^2^Y = 0.98, Q^2^ = 0.963) ion modes, indicating the significant differences in the chemical profile of CHS-J samples produced in the different years. To reveal the discrepant components contributing most to the group separation, the differentiated compounds were picked by *S*-plot derived from the OPLS-DA model constructed by CHS-J samples produced between the year 2014 and year 2018 (Figures 2B,C). Variables with variable importance in the projection (VIP) > 2 were tentatively highlighted to be important for discrimination (Figures 2B,C). The variables were further filtered by *p* < 0.05 and fold-change FC > 2. As a result, 39 and 14 variables were highlighted in positive and negative ion modes, respectively. The structures were tentatively assigned by an in-house database and further validated by their reference standards. Finally, 7 compounds were accurately identified, as shown in Table 1. The intensity tendency of these compounds in CHS-J samples with different storage years was analyzed, and most of the identified compounds had a gradually decreasing trend along with the increasing storage years (data not shown).

**TABLE 1:** Identifications of the highlighted ions which were validated by reference standards.

RT (min)	Formula	[M + H]^+^/[M−H]^-^	Measured mass	Exact mass	Error (ppm)	MS/MS	Compound	Tendency^a^
4.91	C_20_H_24_NO_4_	[M+H]^+^	342.1714	342.1705	2.63	297.1131, 271.0854, 265.0898, 189.0550, 127.0382, 109.0280	Magnoflorine	↓
5.55	C_35_H_46_O_20_	[M−H]^−^	785.2534	785.2504	3.82	623.2162, 161.0267	Echinacoside	↓
11.01	C_38_H_48_O_19_	[M+H]^+^	809.2809	809.2868	−7.29	677.2504, 531.1844, 369.1310, 313.0676	Epimedin B	↓
16.23	C_32_H_38_O_14_	[M+H]^+^	647.2301	647.2340	−6.03	369.1310, 313.0676	Sagittatoside B	↓
16.35	C_33_H_40_O_14_	[M+H]^+^	661.2514	661.2496	2.72	515.1921, 369.1310, 313.0676	2″-O-rhamnosyl Icariside II	↓
16.80	C_27_H_30_O_10_	[M+H]^+^	515.1921	515.1917	0.78	369.1310, 313.0676	Baohuoside Ⅰ	↓
17.92	C_12_H_14_O_2_	[M+H]^+^	191.1069	191.1072	−1.57	173.0959, 145.1019, 115.0564, 105.0679, 91.0563	Z-ligustilide	↓

a CHS-J, produced in the year 2014 vs. 2018.

**FIGURE 3:**
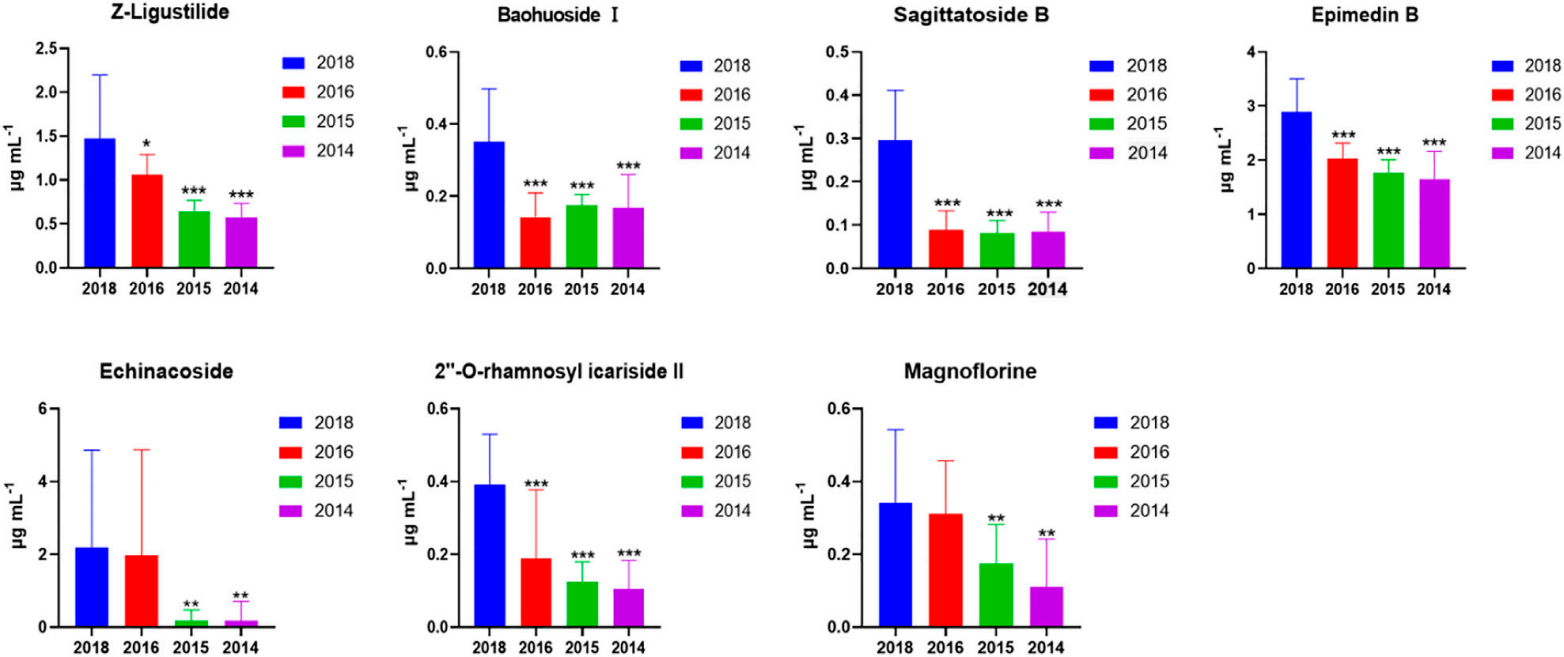
The contents of 7 highlighted components across CHS-J samples produced in different years. Value represents mean ± SD (*n* = 20), *p < 0.05, **p < 0.01, ***p < 0.001 vs. 2018.

To confirm the findings from untargeted metabolomics, these seven highlighted components in CHS-J samples produced in different years were quantitatively determined by UHPLC-QQQ-MS/MS in multiple reaction monitoring (MRM) mode as described previously (Hu et al., 2021). As shown in Figure 3, the concentrations of seven highlighted components across CHS-J samples produced in 2018 were higher than those in tested samples produced in other years, except that no significant difference in the levels of echinacoside or magnoflorine between samples 2018 and 2016.

### Metabolomics Analysis of CHS-J Samples in Accelerated Tests

**FIGURE 4:**
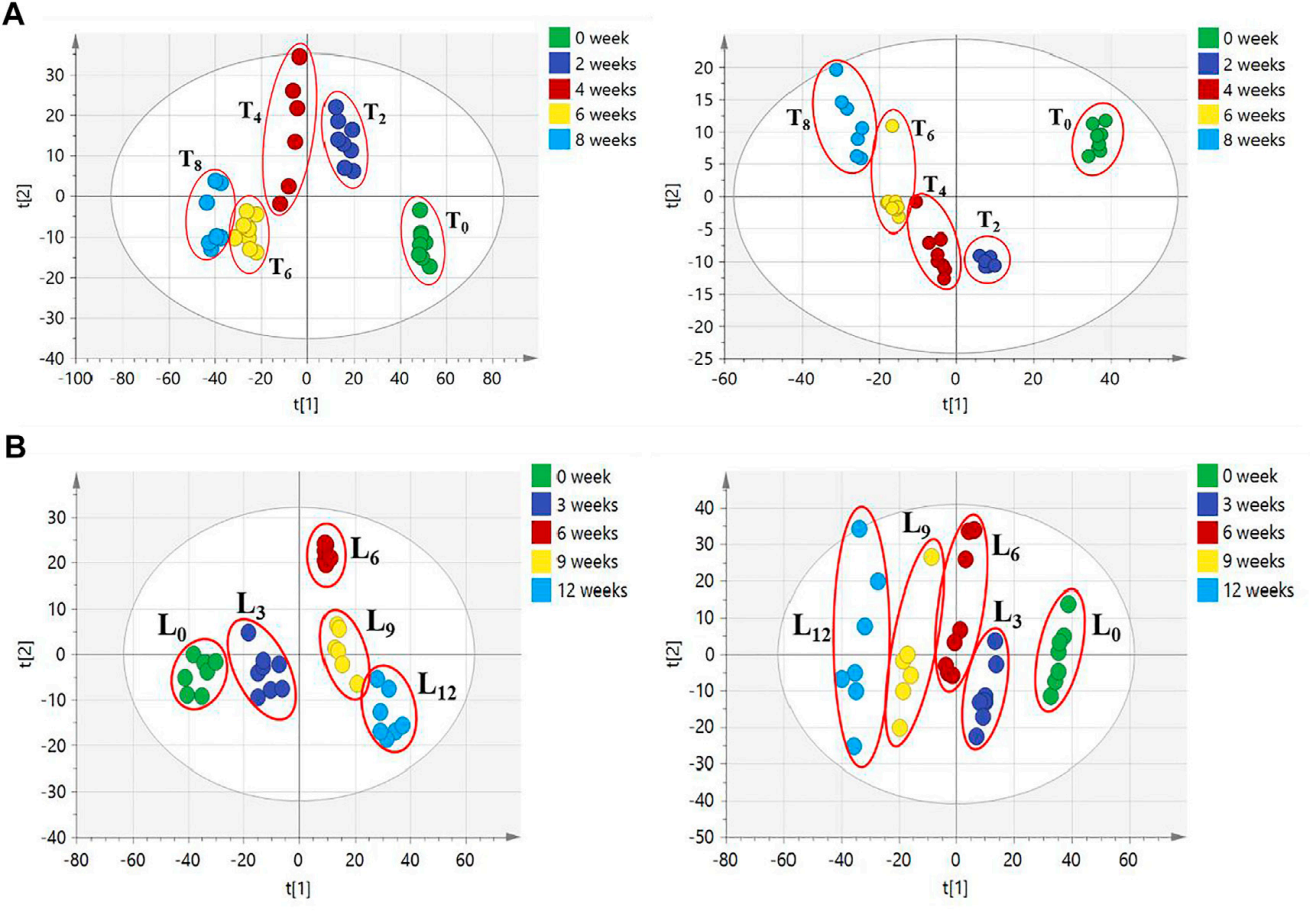
PCA score plots of CHS-J samples in **(A)** temperature- and **(B)** light-accelerated tests detected in positive (left) and negative (right) ion modes.

**FIGURE 5:**
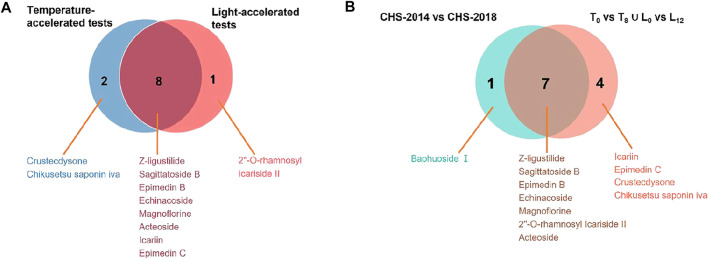
Venn diagrams show the common compounds of **(A)** temperature-accelerated and light-accelerated groups and **(B)** accelerated tests and CHS-J samples produced in different production years.

The difference in these compound levels in CHS-J samples produced in different years might be attributed to the instability of components and the chemical variation of herbal medicines used in different years. To further confirm the instability of components in CHS-J sample, the accelerated tests of the newly produced CHS-J (the year 2020) were conducted. The CHS-J samples were continuously collected for 8 and 12 weeks in temperature-accelerated and light-accelerated tests, respectively. The samples were collected every 2 weeks (T_0_, T_2_, T_4_, T_6_, T_8_) in temperature-accelerated tests and every 3 weeks (L_0_, L_3_, L_6_, L_9_, L_12_) in light-accelerated tests were analyzed by the developed UHPLC-Q-TOFMS. To visualize the chemical profile across groups, the principal component analysis (PCA), an unsupervised data analysis, was conducted. As shown in Figure 4A, all observations were located in the Hotelling T2 (0.95) ellipse, PCA score plots derived from both positive (R^2^X = 0.727, Q^2^ = 0.633) and negative (R^2^X = 0.899, Q^2^ = 0.729) ion modes exhibited tight clusters of CHS-J samples from each time point in temperature-accelerated tests and discrimination across groups. Importantly, the longer treatment by high temperature, the farther the cluster of CHS-J samples departs from untreated CHS-J group (T_0_), indicating the more remarkable changes in the component profile of CHS-J samples along with an increasing treatment time. Similar results were observed in the light-accelerated tests (Figure 4B). To reveal the components contributing most to the group separation, the supervised OPLS-DA of temperature- (T_0_ *vs.* T_8_) and light-accelerated group (L_0_ *vs.* L_12_) were further constructed. The variables with VIP > 2 and *p* < 0.05 were highlighted as important components for the discrimination. As a result, 10 out of 48 and 9 out of 54 differential variables were identified by their reference standards in temperature- (Supplementary Table S2) and light- (Supplementary Table S3) accelerated groups, respectively. Among them, 8 compounds, including Z-ligustilide, echinacoside, magnoflorine, sagittatoside B, epimedin B, icariin, acteoside, and epimedin C, were common features between temperature- and light-accelerated tests (Figure 5A), indicating that these compounds might be sensitive to both temperature and light. While crustecdysone and chikusetsu saponin iva were thermosensitive components, 2′′-O-rhamnosyl Icariside II was photosensitive (Figure 5A). Furthermore, a Venn diagram for CHS-J samples produced by different years and accelerated test datasets show 7 common features (Figure 5B), which indicated that the instability of these components might largely contribute to the differences in CHS-J samples with different storage periods.

### Accelerated Tests of Reference Standards

**FIGURE 6:**
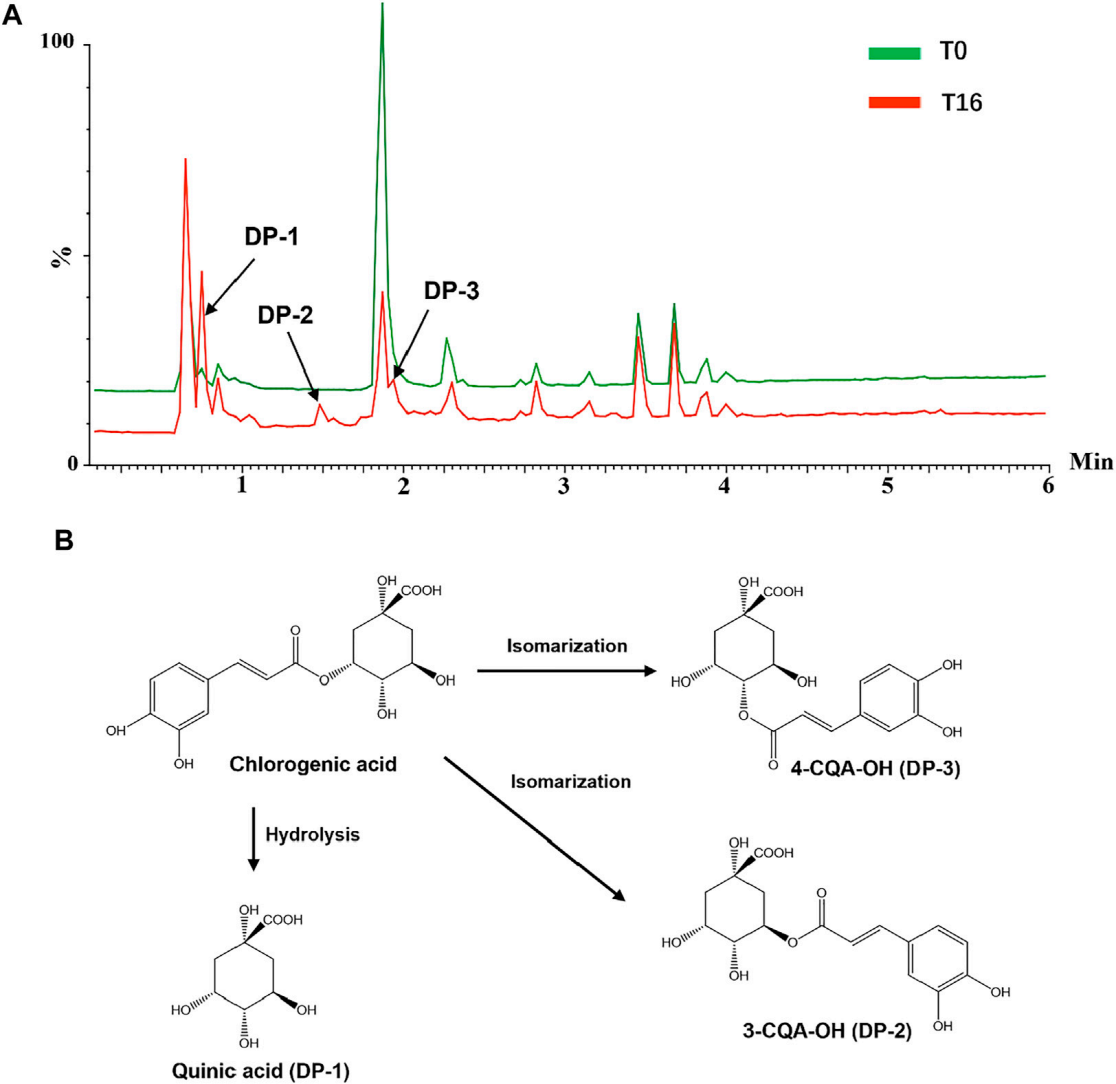
**(A)** TIC of chlorogenic acid dissolved in base liquor before (T_0_) and after 16 weeks (T_16_) in temperature-accelerated tests. **(B)** Proposed degradation mechanism of chlorogenic acid in base liquor under high-temperature exposure.

To further investigate the possible mechanisms underlying these chemical changes, accelerated tests of several reference standards were conducted. The individual reference standards with severe degradation, including chlorogenic acid, echinacoside, baohuoside I, 2″-O-rhamnosyl icariside II, Z-ligustilide dissolved in 35% (v/v) of base liquor were subjected to temperature-accelerated or light-accelerated tests, respectively. The solution before and after accelerated tests were analyzed by a UPLC/Q-TOFMS. The degradation products derived from these reference standards exposed to temperature and light were tentatively assigned by matching their MS and MS/MS data with that of the literatures (Xie et al., 2011; Dawidowicz and Typek, 2010; Zanoelo and Benincá, 2009; Zuo et al., 2013; Yan et al., 2018) and summarized in Supplementary Table S3. Taking chlorogenic acid as an example, the total ion chromatogram (TIC) of chlorogenic acid alcohol base solution (T_0_) and chlorogenic acid alcohol base solution after 16 weeks (T_16_) in temperature-accelerated tests was shown in Figure 6A. Chlorogenic acid was degraded seriously when exposed to high temperatures, and several new products were generated. A newly generated degradation peak at RT = 0.75 min in chlorogenic acid solution after 16 weeks of high-temperature treatment was identified as quinic acid. The presence of quinic acid in alcohol base solution is not surprising because chlorogenic acid is an ester comprised of caffeic and quinic acids. However, caffeic acid was not found in this solution. Two new induced peaks at RT = 1.48 and 1.94 min were identified as 3-O-caffeoylquinic acid and 4-O-caffeoylquinic acid, respectively. The proposed degradation mechanism was speculated and illustrated in Figure 6B. In the presence of hydroxide ion [OH]^−^, the nucleophile on the neighboring hydroxyl group of the quinic acid ring attacked the carbonyl carbon of 5-O-caffeoylquinic acid, which resulted in the internal transesterification of trans-5-caffeoylquinic acid. The caffeoyl moiety transferred from the C5- position to the 4- and 3-positions via ortho-cyclic ester intermediate structures, which further hydrolyzed into esters (Zanoelo and Benincá, 2009; Dawidowicz and Typek, 2010; Xie et al., 2011). But it is difficult to distinguish the two compounds due to their similar MS and MS/MS pattern. Furthermore, chlorogenic acid was also degraded seriously when exposed to light, and its degradation products mainly are the hydroxylated chlorogenic acids (Supplementary Figure S1). Our data indicated that the degradation mechanism of most compounds was hydrolysis and isomerization, which is related to the ethyl alcohol-water environment of CHS-J, while high temperature and light accelerated these degradation processes with varying degrees.

### Anti-Fatigue Effects of CHS-J Samples Produced in 2014 and 2020

Compared to the control group, the exhaustive swimming time, hepatic glycogen level, muscle glycogen level, blood lactic acid level, urea nitrogen level, and lactic dehydrogenase level in both CHS-J samples produced in 2014 and 2020 groups showed a significant difference (*p* < 0.01), except hepatic glycogen and lactic dehydrogenase in CHS-J samples produced in 2020 group (*p* < 0.05), indicating that storage period had little influence on the anti-fatigue effect of CHS-J samples (Table 2). Moreover, compared with CHS-J samples produced in the 2020 group, CHS-J samples produced in the 2014 group had a decreased blood lactic acid and a higher level of lactic dehydrogenase. However, for the exhaustive swimming time, hepatic glycogen level, muscle glycogen level, and urea nitrogen level, there were no significant differences in these four anti-fatigue indexes between CHS-J samples produced in the 2014 group and 2020 group. Collectively, the chemical changes during the storage period had little influence on the anti-fatigue effect of CHS-J samples.

**TABLE 2:** Effects of CHS-J samples produced in 2014 and 2020 on exhaustive swimming time, hepatic glycogen, muscle glycogen, blood lactic acid, urea nitrogen, and lactic dehydrogenase levels in mice (*n* = 10, mean ± SD), *p < 0.05, **p < 0.01 vs. control group, ▲*p* < 0.05 vs. 2014.

Group	Exhaustive swimming time (min)	Hepatic glycogen (mg/g)	Muscle glycogen (mg/g)	Blood lactic acid (mmol/L)	Urea nitrogen (mmol/L)	Lactic dehydrogenase (U/L)
Control	13.20 ± 2.53	6.55 ± 4.56	0.66 ± 0.42	5.82 ± 1.01	8.35 ± 1.29	447.49 ± 64.77
CHS-J (2014)	24.90 ± 3.93**	22.11 ± 8.18**	1.68 ± 0.65**	2.03 ± 0.46**	3.70 ± 0.92**	605.10 ± 90.09**
CHS-J (2020)	22.20 ± 3.08**	18.64 ± 8.26**	1.30 ± 0.49*	3.18 ± 1.19**▲	4.12 ± 0.44**	523.51 ± 86.09*▲

## Conclusion

In this study, the targeted and untargeted metabolomics were integrated to reveal the changed components in different production years of CHS-J. Moreover, accelerated tests were performed to validate the findings in the metabolomics study and explore the stability of compounds in CHS-J. The results demonstrated that some components in CHS-J were changed with the increasing years of storage, and most of them gradually decreased over the duration. The accelerated tests revealed that these changes might be attributed to the chemical degradation of components in CHS-J when exposed to high temperatures or light. Moreover, these changes during the storage period had little influence on the anti-fatigue effect of CHS-J. These findings will offer new insight into understanding the stability of components and anti-fatigue effect in CHS-J and will facilitate the quality control of CHS-J. Meanwhile, the current study indicates that metabolomics is an efficient approach to comprehensively characterize TCM quality in a complex matrix.

## Data Availability

The original contributions presented in the study are included in the article/Supplementary Material, further inquiries can be directed to the corresponding authors.
