# Supplementary material for: Chemical Stability of a Chinese Herbal Spirit Using LC-MS-Based Metabolomics and Accelerated Tests
Source: Front Pharmacol. 2022 Mar 7;13:857706. doi: 10.3389/fphar.2022.857706 (PMC8940302; doi:10.3389/fphar.2022.857706)
Supplement: Supplementary file 1 [file DataSheet1.docx]

Supplementary Material


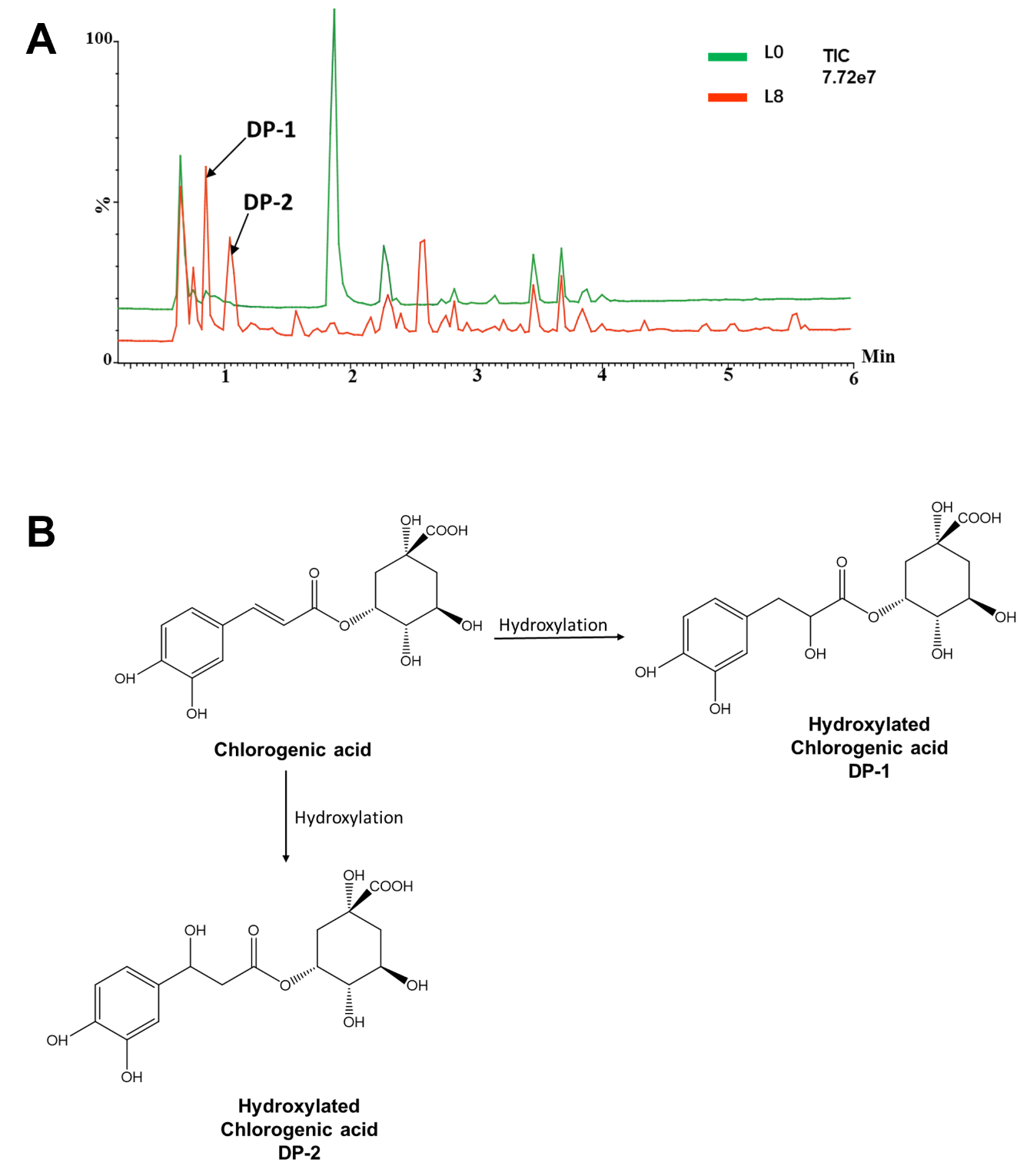


**Supplementary Figure 1.** (A) TIC of chlorogenic acid dissolved in base liquor before (L0) and after 8 weeks (L16) in light-accelerated tests. (B) Proposed degradation mechanism of chlorogenic acid in base liquor under light exposure.

**Supplementary Table 1.** Mass parameters, calibration curves, LOD, LOQ, precision, and recovery of seven investigated analytes in the CHS samples by LC-MS/MS with MRM mode.

| **Analyte** | **RT**  **(min)** | **Transition** | **Cone voltage**  **(V)** | **Collision energy (V)** | **Calibration curve** | | |  | **LOD**  **(ng/mL)** | **LOQ**  **(ng/mL)** |  | **Precision (RSD, %)** | |  | **Recovery**  **(%, n=6)** |
| --- | --- | --- | --- | --- | --- | --- | --- | --- | --- | --- | --- | --- | --- | --- | --- |
|  |  |  |  |  | **Equation** | **R^2^** | **Range**  **(ng/mL)** |  |  |  |  | **Intra-day** | **Inter-day** |  |  |
| Z-ligustilide | 14.73 | 191.11→117.01 | 38 | +22 | y = 8945x - 92 | 0.9998 | 5.4-11000 |  | 0.86 | 2.94 |  | 4.27 | 4.68 |  | 98.8 |
| Baohuoside Ⅰ | 13.83 | 515.30→369.20 | 26 | +20 | y = 48629x + 525 | 0.9988 | 0.4-3375 |  | 0.03 | 0.10 |  | 0.99 | 2.69 |  | 96.8 |
| Sagittatoside B | 13.45 | 647.45→369.22 | 24 | +24 | y = 9750x + 95 | 0.9998 | 3.5-3600 |  | 0.59 | 2.34 |  | 4.79 | 4.88 |  | 109.1 |
| Epimedin B | 9.31 | 809.57→369.22 | 30 | +34 | y = 7352x + 1340 | 0.9924 | 6.7-13800 |  | 0.19 | 0.75 |  | 1.08 | 3.85 |  | 99.4 |
| Echinacoside | 4.91 | 787.55→325.10 | 20 | +20 | y = 317.6x + 5.9 | 0.9995 | 460-11778 |  | 2.16 | 7.66 |  | 3.38 | 3.51 |  | 90.9 |
| 2''-O-rhamnosyl Icariside II | 13.51 | 661.47→369.28 | 28 | +21 | y = 14713x + 522 | 0.9979 | 2.5-5150 |  | 0.11 | 0.38 |  | 3.47 | 4.15 |  | 90.6 |
| Magnoflorine | 4.45 | 342.30→297.30 | 42 | +19 | y = 27481x + 1247 | 0.9992 | 1.4-5542 |  | 0.25 | 0.74 |  | 1.87 | 3.99 |  | 104.8 |

**Supplementary Table 2.** The identifications of the highlighted ions by their reference standards in temperature-accelerated tests.

| **RT (min)** | **Formula** | **[M + H]^+^**  **/ [M - H]^-^** | **Measured mass** | **Exact mass** | **Error (ppm)** | **MS/MS** | **Compound** | **Tendency** |
| --- | --- | --- | --- | --- | --- | --- | --- | --- |
| 5.01 | C_20_H_24_NO_4_ | [M＋H] ^+^ | 342.1672 | 342.1705 | -9.64 | 297.0899, 265.0674, 137.0503 | Magnoflorine | ↓ |
| 5.63 | C_35_H_46_O_20_ | [M－H] ^-^ | 785.2500 | 785.2504 | -0.51 | 623.2230, 161.0260 | Echinacoside | ↓ |
| 6.96 | C_27_H_44_O_7_ | [M＋H] ^+^ | 481.3190 | 481.3165 | 5.19 | 445.2963, 427.2836, 371.2264 | Crustecdysone | ↓ |
| 7.11 | C_29_H_36_O_15_ | [M－H] ^-^ | 623.1925 | 623.1976 | -8.18 | 623.0255, 462.0724, 161.0664 | Acteoside | ↓ |
| 11.00 | C_38_H_48_O_19_ | [M＋H] ^+^ | 809.2875 | 809.2868 | 0.86 | 677.2391, 531.1869, 369.1355, 313.0726 | Epimedin B | ↓ |
| 11.22 | C_39_H_50_O_19_ | [M＋H] ^+^ | 823.3002 | 823.3024 | -2.67 | 531.1472, 369.1067, 313.0476 | Epimedin C | ↓ |
| 11.45 | C_33_H_40_O_15_ | [M＋H] ^+^ | 677.2450 | 677.2446 | 0.59 | 531.1472, 369.1067, 313.0476 | Icariin | ↑ |
| 15.16 | C_42_H_66_O_14_ | [M－H] ^-^ | 793.4309 | 793.4374 | -8.19 | 631.3848 | Chikusetsu saponin iva | ↑ |
| 16.21 | C_32_H_38_O_14_ | [M＋H] ^+^ | 647.2393 | 647.2340 | 8.19 | 531.1527, 369.1067, 313.0476 | Sagittatoside B | ↓ |
| 17.98 | C_12_H_14_O_2_ | [M＋H]^+^ | 191.1056 | 191.1072 | -8.38 | 173.0804, 135.0340, 113.9567, 108.8926 | Z-ligustilide | ↓ |

**Supplementary Table 3.** The identifications of the highlighted ions by their reference standards in light-accelerated tests.

| **RT (min)** | **Formula** | **[M + H]^+^**  **/ [M - H]^-^** | **Measured mass** | **Exact mass** | **Error (ppm)** | **MS/MS** | **Compound** | **Tendency** |
| --- | --- | --- | --- | --- | --- | --- | --- | --- |
| 5.02 | C_20_H_24_NO_4_ | [M＋H]^+^ | 342.1685 | 342.1705 | -5.84 | 297.1107, 265.0810 | Magnoflorine | ↓ |
| 5.65 | C_35_H_46_O_20_ | [M－H]^-^ | 785.2450 | 785.2504 | -6.88 | 623.2122 | Echinacoside | ↓ |
| 7.13 | C_29_H_36_O_15_ | [M－H]^－^ | 623.2012 | 623.1976 | 5.78 | 161.0204 | Acteoside | ↓ |
| 11.00 | C_38_H_48_O_19_ | [M＋H]^+^ | 809.2865 | 809.2868 | -0.37 | 677.2391, 531.1869, 369.1355, 313.0726 | Epimedin B | ↓ |
| 11.22 | C_39_H_50_O_19_ | [M＋H]^+^ | 823.3033 | 823.3024 | 1.09 | 677.2391, 531.1869, 369.1355, 313.0726 | Epimedin C | ↓ |
| 11.45 | C_33_H_40_O_15_ | [M＋H]^+^ | 677.2463 | 677.2446 | 2.51 | 531.1869, 369.1355, 313.0726 | Icariin | ↓ |
| 16.22 | C_32_H_38_O_14_ | [M＋H]^+^ | 645.2176 | 645.2183 | -1.08 | 367.1178 | Sagittatoside B | ↓ |
| 16.33 | C_33_H_40_O_14_ | [M－H]^－^ | 659.2333 | 659.2340 | -1.06 | 367.1178 | 2′'-O-rhamnosyl Icariside II | ↓ |
| 17.98 | C_12_H_14_O_2_ | [M＋H]^+^ | 191.1091 | 191.1072 | 9.94 | 149.9312, 135.1178, 110.9514, 94.7847 | Z-ligustilide | ↓ |

**Supplementary Table 4.** Identifications of degradation products derived from reference standards exposure to temperature and light.

| **Compound** | **Accelerated Tests** | **Detection mode** | **RT (min)** | **MS** | **MS/MS** | **Elemental composition** | **Identification** |
| --- | --- | --- | --- | --- | --- | --- | --- |
| Chlorogenic acid | Temp. | [M - H]^-^ | 1.87 | 353.0845 | 191.0558 | C_16_H_18_O_9_ | Chlorogenic acid |
|  |  |  | 0.75 | 191.0558 | 127.0405, 93.0329, 85.0305 | C_7_H_12_O_6_ | Quinic acid |
|  |  |  | 1.48 | 353.0845 | 243.8974, 191.0558, 179.0363, 146.9627, 135.0454 | C_16_H_18_O_9_ | 3-O-caffeoylquinic acid |
|  |  |  | 1.94 | 353.0845 | 191.0558, 179.0363, 173.0436, 135.0454, 93.0329 | C_16_H_18_O_9_ | 4-O-caffeoylquinic acid |
| Baohuoside Ⅰ | Temp. | [M - H]^-^ | 7.75 | 513.1866 | 366.1141 | C_27_H_30_O_10_ | Baohuoside Ⅰ |
|  |  |  | 9.80 | 367.1178 | 309.0367, 297.0378 | C_21_H_20_O_6_ | Icaritin |
|  |  |  | 5.29 | 531.1836 | 384.1251, 369.0957, 341.1054, 243.8974 | C_27_H_32_O_11_ | Hydroxylated Baohuoside Ⅰ |
| 2''-O-rhamnosyl icariside II | Temp. | [M - H]^-^ | 7.23 | 659.2359 | 366.1141 | C_33_H_40_O_14_ | 2''-O-rhamnosyl icariside II |
|  |  |  | 5.05 | 677.2465 | 661.1777, 243.8974, 225.9242 | C_33_H_42_O_15_ | Hydroxylated 2''-O-rhamnosyl icariside II |
|  |  |  | 9.79 | 367.1178 | 309.0367, 281.0425 | C_21_H_20_O_6_ | Icaritin |
|  |  |  | 7.72 | 513.1767 | 366.1141, 325.1843, 243.8974 | C_27_H_30_O_10_ | Baohuoside Ⅰ |
| Z-ligustilide | Light | [M + H]^+^ | 9.03 | 191.1126 | 173.0959, 145.1023, 117.0696 | C_12_H_14_O_2_ | Z-ligustilide |
|  |  |  | 2.93 | 247.0923 | 207.1029, 189.0907 | C_12_H_16_O_4_ | (E)-6, 7-dihydroxyligustilide |
|  |  |  | 3.11 | 247.0923 | 207.1029, 189.0907 | C_12_H_16_O_4_ | Senkyunolide I |
|  |  |  | 4.13 | 247.0923 | 207.1029, 189.0907 | C_12_H_16_O_4_ | Senkyunolide H |
|  |  |  | 10.09 | 403.1846 | 381.2068, 191.1066 | C_24_H_28_O_4_ | Z,Z‘-6.8’,7.3‘-Diligustilide |
|  |  |  | 10.55 | 403.1846 | 381.2068, 191.1066 | C_24_H_28_O_4_ | Angelicide |
|  |  |  | 10.71 | 403.1846 | 381.2068, 191.1066 | C_24_H_28_O_4_ | Levistolide A |
|  |  |  | 10.90 | 403.1846 | 381.2068, 191.1066 | C_24_H_28_O_4_ | Z,Z‘-3.3’,8.8‘-Diligustilide |
| Echinacoside | Light | [M - H]^-^ | 2.40 | 785.2573 | 623.2122, 161.0260 | C_35_H_46_O_20_ | Echinacoside |
|  |  |  | 1.17 | 623.2230 | 623.2230 | C_29_H_36_O_15_ | Acteoside |
|  |  |  | 1.87 | 803.2679 | 773.2462, 623.2122 | C_35_H_47_O_21_ | Hydroxylated echinacoside |
|  |  |  | 2.13 | 803.2679 | 773.2462, 623.2122 | C_35_H_47_O_21_ | Hydroxylated echinacoside |
| Chlorogenic acid | Light | [M - H]^-^ | 1.87 | 353.0845 | 191.0558 | C_16_H_18_O_9_ | Chlorogenic acid |
|  |  |  | 0.85 | 371.0957 | 341.0893, 339.0673, 191.0558, 173.0436, 135.0454 | C_16_H_20_O_10_ | Hydroxylated chlorogenic acid |
|  |  |  | 1.04 | 371.0957 | 341.0893, 339.0673, 191.0558, 173.0436, 135.0454 | C_16_H_20_O_10_ | Hydroxylated chlorogenic acid |
